# Strategies for teaching evidence-based practice in nursing education: a thematic literature review

**DOI:** 10.1186/s12909-018-1278-z

**Published:** 2018-07-28

**Authors:** May-Elin T. Horntvedt, Anita Nordsteien, Torbjørg Fermann, Elisabeth Severinsson

**Affiliations:** 1 Faculty of Health and Social Sciences, the Department of Nursing and Health Sciences, University of South-Eastern Norway, P.O. Box 235, N-3603 Kongsberg, Norway; 2 Department of Research and Internationalisation, University of South-Eastern Norway, P.O. Box 235, N-3603 Kongsberg, Norway; 3 Centre for Women’s, Family and Child Health, Faculty of Health and Social Sciences, University of South-Eastern Norway, P.O. Box 235, N-3603 Kongsberg, Norway

**Keywords:** Teaching strategies, Evidence-based practice, Nursing education, Curriculum, Learning outcome

## Abstract

**Background:**

Evidence-based practice (EBP) is imperative for ensuring patient safety. Although teaching strategies to enhance EBP knowledge and skills are recommended, recent research indicates that nurses may not be well prepared to apply EBP. A three-level hierarchy for teaching and learning evidence-based medicine is suggested, including the requirement for interactive clinical activities in EBP teaching strategies. This literature review identifies the teaching strategies for EBP knowledge and skills currently used in undergraduate nursing education. We also describe students’ and educators’ experiences with learning outcomes and barriers.

**Methods:**

We conducted literature searches using Medline, Embase, CINAHL, ERIC and Academic Search Premier. Six qualitative studies and one mixed-method study met the inclusion criteria and were critically evaluated based on the Critical Appraisal Skills Programme. Using Braun and Clarke’s six phases, the seven studies were deductively and thematically analysed to discover themes.

**Results:**

Four teaching strategy themes were identified, including subthemes within each theme: i.e., interactive teaching strategies; interactive and clinical integrated teaching strategies; learning outcomes; and barriers. Although four studies included a vague focus on teaching EBP principles, they all included research utilisation and interactive teaching strategies. Reported learning outcomes included enhanced analytical and critical skills and using research to ensure patient safety. Barriers included challenging collaborations, limited awareness of EBP principles and poor information literacy skills.

**Conclusion:**

Four of the seven analysed studies included a vague focus on the use of EBP teaching strategies. Interactive teaching strategies are used, but primary strategies focus on searching for and critically appraising research for practice-based application. Although this review included a relatively small sample of literature, the findings indicate a need for more qualitative research investigating interactive and clinically integrated teaching strategies towards further enhancing EBP undergraduate nursing students’ knowledge and skills.

## Background

Evidence-based practice (EBP) in health care has become imperative for patient safety. EBP involves a conscious use and application of various knowledge sources, including the use of published research in conjunction with clinical expertise and patient values and preferences [1]. The process of EBP includes that health-care personnel formulate structured queries, and then conduct searches of databases from which they acquire trustworthy and reliable evidence. Further, they must then critically appraise the research for its reliability, validity and applicability to a clinical context [2, 3].

Interactive methods including interactive lectures, small group work, journal clubs, reading quizzes, clinical nurse presentations, workshops and problem-based learning are needed in teaching EBP [2, 3]. An interactive approach involves an interaction amongst the participants [3]. Effective learning reflects the quality of teaching. Learning though a constructivist approach refers to the creation of an environment in which the learner is an active participant who gains experience and engages in reflection, leading to problem-based, transformative learning [4].To engage the next generation of nurses and enhance their EBP knowledge and skills, a variety of teaching strategies have been recommended [5–7].

Khan and Coomarasamy [3] have described a three-level hierarchy of evidence-based medicine (EBM) teaching and learning methods. The first level is interactive clinical activities. The second level is classroom didactics using clinical and interactive activities. Finally, although less preferred for teaching EBP, the third level is classroom didactic or stand-alone teaching [3]. According to Fineout-Overholt et al. [2], it is important to keep teaching strategies simple and integration of EBP must be a natural part of the academic culture.

Research supports the first level in this hierarchy when teaching EBP; i.e., an interactive style is often preferred because this method facilitates student learning [8–10]. Johnson et al. [11] found that new learning methods and blended approaches to teaching EBP impact students’ attitudes towards research. In addition, Crookes et al. [12] identified different meaningful and engaging teaching strategies that have been adopted by nurse educators, such as online teaching, gaming and simulation techniques. However, these authors also concluded that nursing education needs to include more active lecture styles to strengthen the link between course content and clinical practice [12]. Ryan [10] introduced teaching strategies as extrinsic factors and found that teaching EBP and research methods may be more relevant if taught in a clinical context rather than using traditional didactic methods.

A mixed-methods meta-synthesis examining awareness and adoption of EBP stated that EBP skills for registered nurses and Bachelor of Science in Nursing (RN-to-BSN) students are influenced by exposure to partnerships and contextual teaching and learning, as well as clinical practice experience [13]. Teaching and learning strategies have included clinical practicum projects, lectures, small group work, post-clinical conferences, online modules and simulations [13]. EBP teachers who collaborate with their students, and nurses in clinical practice also influence students’ integration of EBP [2].

To ensure nursing students’ enhanced EBP knowledge, it is also essential to build partnerships with librarians who teach information literacy, which involves searching for relevant research in databases and evaluating and using that information in relation to course requirements and assignments [2, 14]. Reported barriers to the adoption of EBP include difficulties with searching databases and evaluating research, feeling isolated from knowledgeable colleagues and the perception that there are minimal benefits from EBP. Countering these barriers, Phillips and Cullen [13] found that a variety of teaching and learning strategies may empower students’ implementation of EBP in clinical practice.

Emerson and Records’ [15] overview of scholarship and its role in nursing education includes a description of catalysts that enhance EBP in nursing and the knowledge necessary for EBP teaching. They state that scholarly teaching is an academic expectation; however, it does not appear to advance either the education or the discipline beyond the individual level. Indeed, nurses face challenges to EBP from their inability to locate and critically evaluate information [16–19].

The European Higher Education Area (EHEA) framework specifies expected learning outcomes for candidates with a Bachelor’s degree, including skills in finding, evaluating, referring and applying scientific information [20]. Likewise, the Code of Ethics of the International Council of Nurses stresses that nurses must be aware of and implement research results into their clinical practice [21]. Despite these guidelines, it appears that teaching EBP in nursing education varies among nurse educators and universities, and that clinical preceptors may have insufficient knowledge needed to support students [2, 10, 19]. Recent research indicates that nurses may not be well prepared to use EBP in their clinical practice [22, 23].

There is a dearth of literature regarding the effect of teaching and learning strategies on implementing EBP in nursing education [10, 13, 23, 24] and it is currently unclear whether implementation of EBP training leads to improved nursing practice [13].

### Aim

In this literature review, we aimed to identify strategies for teaching EBP in undergraduate nursing education. The review questions were: “What teaching strategies are used to enhance knowledge and skills in EBP in undergraduate nursing education and what are the learning outcomes and barriers?”

## Methods

### Identification of studies

We conducted literature searches using Medline, Embase, CINAHL, Academic Search Premier and ERIC. The PICo framework for qualitative research was used to develop the review questions, plan the search and define the inclusion criteria. The population or participants assessed were nursing students, nursing education and nursing programmes. The phenomenon of interest was teaching and the specific context was EBP education. These concepts were transformed into the actual subject headings and text used in the search strategy in Medline (Table 1), which represents how the concepts were truncated and combined using Boolean and proximity operators in all database searches. The search criteria included qualitative studies published in English from 2006 through 2017. This range was chosen based on an initial search in PubMed PubReMiner indicating that most research on EBP training in nursing education was published since 2006, when EBP gained a foothold in nursing education. We examined the references cited in the retrieved studies, as well as studies in Google Scholar that cited the retrieved studies.

**Table 1:** Example of the search strategy in Medline

1. exp. Education, Nursing/	
2. (nurs* adj3 education).ab, ti.	
3. (nurs* adj3 program*).ab, ti.	
4. (nurs* adj3 student*).ab, ti.	
5. 1 or 2 or 3 or 4	
6. teaching.ab, ti.	
7. exp. Teaching/	
8. 6 or 7	
9. exp. Evidence-Based Practice/	
10. evidence-based.ab, ti.	
11. 9 or 10	
12. 5 and 8 and 11	
13. limit 12 to (english language and yr. = “2006–2017”)	
14. limit 13 to “qualitative (best balance of sensitivity and specificity)”	

The inclusion criteria were: 1) original, qualitative research focused on EBP teaching strategies in undergraduate nursing education, i.e., we focused on qualitative research to gain a deeper insight into teacher and student experiences with these strategies; 2) peer-reviewed, original research; 3) studies on educators, student participation, or both; and 4) studies evaluated as moderate or high quality according to the Critical Appraisal Skills Programme (CASP) [25]. The exclusion criteria were: reviews, quantitative studies, theoretical studies and contributions that were not original research articles. Articles related to teaching strategies directed at health-care personnel, master programmes or postgraduate nursing education were also excluded.

**Fig. 1:**
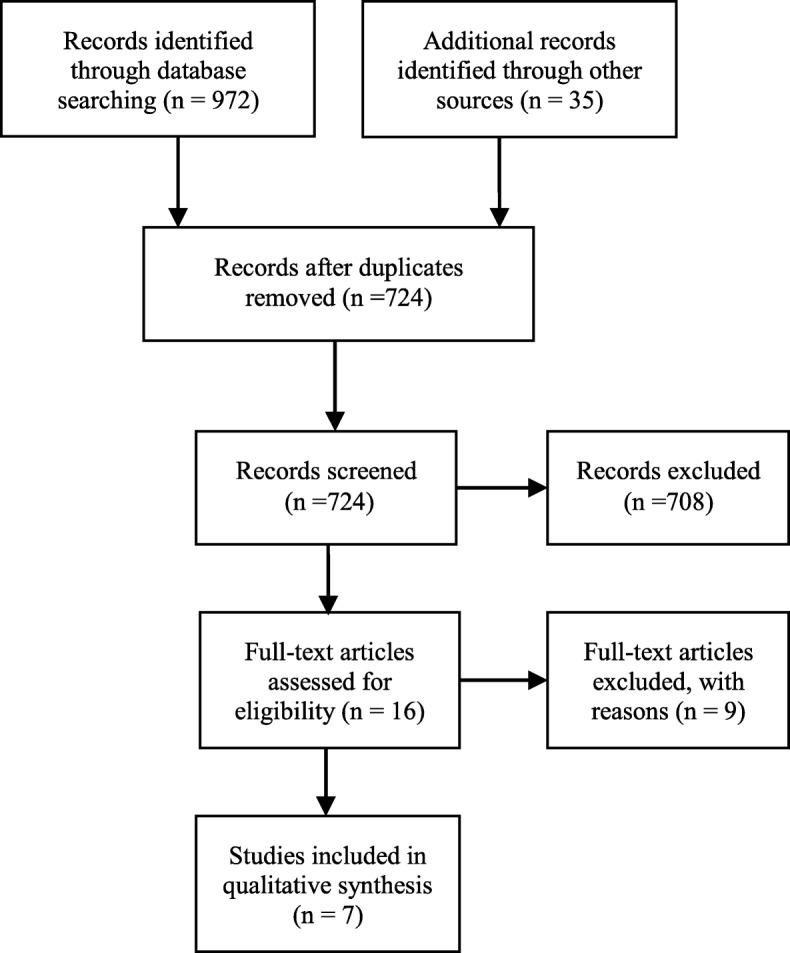
PRISMA flowchart of the screening and the assessment process

We used the Preferred Reporting Items for Systematic Reviews and Meta-Analysis (PRISMA) [26] flowchart in the retrieval and selection process (Fig. 1) to identify 972 records from an initial database search and an additional 35 by manually searching those studies’ bibliographies. After duplicates were eliminated, we screened the abstracts of 724 articles. Of these, 708 articles did not meet our inclusion criteria, thus we obtained 16 full-text articles for further analysis. Each of the four authors examined all 16 articles, of which nine were excluded because of their low quality, focus on clinical intervention, or lack of focus on undergraduate nursing education. The final seven articles were included in the review.

### Critical appraisal

All four authors independently appraised the seven final articles for their methodological quality using CASP (Table 2), with moderate and high methodological quality defined as meeting 6–8 and 9–10 of the CASP checklist criteria, respectively. We discussed disagreements until consensus was reached.

**Table 2:** Quality assessment based on the CASP Qualitative Research Checklist

	Authors	1	2	3	4	5	6	7	8	9	10	Assessment
1	André et al. [28]	Y	Y	Y	Y	U	N	Y	U	Y	Y	Moderate
2	Cader et al. [29]	Y	Y	Y	N	Y	Y	Y	Y	Y	U	Moderate
3	Friberg and Lyckhage [30]	Y	Y	Y	Y	Y	N	N	U	Y	U	Moderate
4	Irvine et al. [34] Mixed methods	Y	Y	Y	U	Y	N	Y	U	Y	Y	Moderate
5	Malik et al. [31]	Y	Y	Y	Y	Y	U	Y	Y	Y	Y	High
6	Mattila and Eriksson [32]	Y	Y	Y	Y	Y	Y	Y	Y	Y	Y	High
7	Nayda and Rankin [33]	Y	Y	Y	Y	Y	N	Y	Y	Y	Y	High

*CASP* criteria for qualitative studies: 1. Was there a clear statement of the aims of the research?; 2. Was a qualitative methodology appropriate?; 3. Was the research design appropriate to address the aims of the research?; 4. Was the recruitment strategy appropriate to the aims of the research?; 5. Was the data collected in a way that addressed the research issue?; 6. Has the relationship between researcher and participants been adequately considered?; 7 Have ethical issues been considered?; 8. Was the data analysis sufficiently rigorous?; 9. Is there a clear statement of the findings?; 10. How valuable is the research? (*Y* Yes, *N* No, *U* Unclear)

### Analysis

A thematic analysis was conducted to identify themes based on the six phases described by Braun and Clarke [27], whose deductive approach refers to themes identified top down; in other words, we coded themes based on our specific review question. Although Braun and Clarke [27] recommend using narrative text, the included qualitative studies and mixed-methods study provided text-based data. In the first phase, all four authors familiarised themselves with the research by reading and rereading the data from each study. In the second phase, the first author carried out a systematic, manual coding of features that led to initial codes, before searching for themes in the third phase. Phase four involved reviewing the themes for correlation with the codes and identification of subthemes. After defining the themes in phase five, the findings were evaluated for relevance to the research question. The authors met several times to discuss the analysis process and to reach consensus on the labelling.

## Results

A summary of the studies and their findings are presented in Table 3. The seven studies were conducted in Norway, the United Kingdom (UK), Sweden, Australia and Finland [28–33]. Qualitative data were also gathered from one mixed-methods study [34] conducted in the UK which, although using mixed methods, reported qualitative findings from students’ graffiti board comments and a focus group interview regarding lectures.

**Table 3:** Studies included in this review

Authors, country, journal	Aim	Methodology	Summary of findings
André et al. [28] Norway *Nurse Education in Practice*	How undergraduate students can increase skills and knowledge towards EBP through participation in clinical research projects.	Pilot: Qualitative Sample: Five third-year nursing students Data collection: qualitative data from open-ended questions in a questionnaire. Kvale’s analysis method.	Four-stage intervention: 1. Information about voluntary participation in clinical research projects; 2. Education programme related to EBP; 3. Participation in clinical research projects; 4. Instructions and education on analysing and discussing findings. Allocating nursing students to small research projects and allowing them to participate in research projects is useful. Students experienced lack of EBP knowledge when joining the study. Positive collaboration with nurses. While students were motivated individually, external motivational factor was essential. The experience led to a positive approach and EBP knowledge.
Cader et al. [29] UK *Nurse Education Today*	Pre-registration nursing programme: exploring student experiences with accessing and utilising information for an assignment aimed at enhancing EBP skills.	Qualitative Sample: 16 nursing students Data collection: two focus groups. Grounded theory used in the analysis process.	Teaching strategy: conducting health needs analyses through collaboration with practice-based assignment. Three main themes were identified: 1. Challenges in evidence gathering: i.e., students need more skills in accessing, utilising and appraising information; 2. Nature of support needed: guidance from academic and library staff working in collaboration is required to enhance EBP skills; 3. Understanding the importance of evidence for practice: Bridging the gap between theory and practice. Development of computer and information literacy (IL) skills is important.
Friberg and Lyckhage [30] Sweden *Nursing Education Perspectives*	Development of literature-based models for Bachelor’s degree essays and evaluation of students’ experiences.	Qualitative Action Sample: 34 lecturers and nearly 80 students divided into seven student groups who participated over the course of 4 years. Data collection: Informal interviews and field notes. Reflective journals of 86 students. Content analysis.	Teaching strategy: writing Bachelor’s degree, essays and establishing teaching modules related to the research process (concept analysis, identifying evidence-based quantitative and qualitative research, critical assessment, identifying discourses in documents and practical implications of research). Cross-professional collaboration between faculty and library to improve lecturers’ competence. Literature-based models provided guidance to students and improved their skills in literature retrieval and academic writing. Development of computer literacy and IL skills.
Irvine et al. [34] UK *Nurse Education in Practice*	Implementation of changes to improve the teaching and learning of research methods in a second-year pre-registration nursing programme at one university. Evaluation of students’ and lecturers’ experiences.	Mixed methods 1. A questionnaire answered by 49 out of 53 nursing students. 2. An informal qualitative ‘graffiti board’ evaluation of the cohort. 3. Group interview with four lecturers. Calculation of mean questionnaire scores and framework analysis.	Teaching strategy: Established teaching modules related to the research process through student-centred approaches: e.g., experiential learning supported by the university’s virtual learning platform. Teaching and learning approaches: teaching sessions, guided sessions, group work and presentation. Conducting a small research project in groups facilitated by a lecturer: conducting a literature review, developing a proposal, ethics, collecting and analysing data and presenting results. Outcome: research understanding, critical analysis and poster presentations. EBP and literacy as an integrated element throughout the whole curricula (faculty and library). Different dispositions towards learning. Important to develop good learning environments.
Malik et al. [31] Australia *Journal of Advanced Nursing*	Understand the processes used by nurse academics when integrating EBP into teaching and learning.	Qualitative: constructivist grounded theory approach Sample: 23 Australian academic nurses. Interviews, nine participants observed while teaching. Document analysis.	Nurse academics are practicing diverse teaching and learning strategies, including lectures, tutorials, laboratory work, online activities and assignments. Some use the latest evidence in their lectures. Clinical experts are invited to give lectures. Teaching approaches, such as facilitating students’ learning by asking clinical questions, are used. Others teach EBP knowledge in laboratories. Academics perceive it to be challenging to find motivating and innovative teaching approaches. Different barriers such as lack of time, knowledge and experience with innovative teaching approaches were identified. Students seem to enjoy database-searching workshops delivered by the library staff. The first-year students learned to search for research and in their third year, students expected to conduct critical analysis research. A few academics strived to include the EBP process in the practice context. Through lectures, academics attempted to contextualise EBP with an aim to link evidence to practice.
Mattila and Eriksson [32] Finland *Nurse Education Today*	Examine the significance of a learning assignment in relation to research skills and nursing students’ learning in clinical practice.	Qualitative: descriptive Sample: 50 nursing students in clinical practice. Data collection: six open-ended questions related to research skills, learning during clinical practice and further development of the assignment. Content analysis.	Teaching strategy: Six-week programme in reading and oral presentations of results from a research article related to a chosen field and topic of nursing practice. Learning research skills created a better understanding of the format and core of an article in addition to introducing new perspectives. Oral presentation helped clarify research concepts, creativity was shown in presentations, increased awareness of research findings and clinical practice, academic presentation and discussion inspired the search for further research. The programme broadened students’ understanding of nursing care and their future nursing role, nurses’ responsibilities and evaluation of nursing outcomes.
Nayda and Rankin [33] Australia *Australian Journal of Advanced Nursing*	Map development of IL skills among BN students. Evaluate BN students’ and academics’ understandings of IL, IL links to lifelong learning and subsequent implications for high-quality EBP.	Qualitative, exploratory and triangulated Sample: focus group of 394 BN students and seven academics. Data collection: combination of document analysis, questionnaire and focus groups. Thematic analysis.	The main themes were roles (of library, lectures and student peers) and collaborative strategies in the curriculum. Despite completing an IL course, students lacked a comprehensive understanding of IL and IL skills, while academics related the term ‘IL’ to general literacy and had varying levels of computer knowledge. Study outcomes indicated the need for staff development and a progressive approach to the curriculum to ensure that students understand IL and its links to life-long learning, which requires collaboration between librarians, study skills advisors and academics. There is a need for an orchestrated and progressive process to ensure development of students’ IL skills, computer and information literacy, EBP and literacy as an integrated element throughout the whole curricula as well as cross-professional collaboration (faculty and library) in teaching.

The four themes (and subthemes within each theme) were: 1) Interactive teaching strategies (Research utilisation, Information literacy and Assignments as learning activities); 2) Interactive and clinically integrated teaching strategies (Teaching EBP principles and Clinical integration and collaborations); 3) Learning outcomes (Enhancing analytical skills and Changing attitudes toward utilising research); and 4) Barriers (Information literacy skills and knowledge and Challenging collaboration).

### Interactive teaching strategies

An improved understanding of the differences between quantitative and qualitative methods was highlighted as an important aspect of preparation for nursing practice [29–32, 34]. Interactive strategies to teach the research process, critical appraisal and development of information literacy skills were also emphasised. Interactive learning activities such as problem-based learning, sharing information, flipped classroom and virtual simulation, workshops, group work and seminars with discussions were identified [30, 33, 34]. In some studies, oral presentations of students’ research findings in a clinical setting were highlighted as an important part of the teaching and learning strategy [28, 32, 34].

#### Research utilisation

Traditional teaching methods preparing students to use research were aimed at improving critical thinking skills, critically evaluating various literature sources and developing information literacy skills [30–32, 34].

Group work was also identified as a teaching strategy for establishing research utilisation [28, 30, 34]. In the mixed-methods study [30], several workshops and monthly sessions were conducted to improve research competence among both lecturers and students.

Experiential learning was often supplemented by collaborative group learning, such as partnerships for learning course content [34]. The authors presented experiential teaching approaches as a motivational tool for improving research learning. The students used student-centred approaches and completed small group research studies. Assignments included carrying out a literature review, developing a proposal, facing a mock ethics committee, and collecting and analysing data. This student work was supported by pertinent lectures, including via ‘Blackboard’, a virtual learning platform. Finally, students presented their methodologic and analytic approaches on the virtual learning platform [34].

#### Information literacy

Teaching information literacy and interdisciplinary collaboration, especially with librarians, was emphasised as an important part of students’ learning how to find and use research [29–31, 33–35]. In contrast, collaboration with librarians was not mentioned in Mattila and Eriksson’s [32] study.

Friberg and Lyckhage’s [30] study emphasised the significance of research utility and disseminating research results. Cader et al. [29] revealed differences in students’ knowledge of computer and information literacy skills. In one study, differences appear to have been influenced by the role of the library in supporting nursing students, curriculum content and emphasis, and interaction with lecturers and peers [33]. Nurse educators indicated a need for staff development and a progressive approach to the curriculum to ensure students’ understanding of IL and its links to learning [33].

An environment supportive of the learning process promoted change and development. Collaboration with and facilitation and guidance by academic and library staff was considered essential for a successful process and outcome [29, 33]. It was also clear from these studies that nursing students need greater support to access, use and evaluate information fully [29, 32, 33].

#### Assignments as learning activities

Course assignments were included as a part of the learning process in all evaluated studies, which included activities preparing students to use research or enhance their EBP knowledge and skills. Assignments that were integrated into clinical practice were particularly emphasised in the studies that focused on teaching EBP principles [28, 29, 31].

An assignment focusing on analysis of health needs was undertaken to help student nurses gain an understanding of the relevance of EBP [29]. Nursing students were found to require further development of their critical appraisal skills and further improvement of the guidance from both academics and librarians was needed [29].

Friberg and Lyckhage [30] emphasised essay writing as a learning tool and used different literature-based research methods to meet this goal.

### Interactive and clinically integrated teaching strategies

The thematic analysis identified interactive and clinically integrated teaching strategies. Interactive clinical strategies included assignments based on collaboration with health-care personnel in clinical practice. Learning activities with oral presentations of the findings from the students’ studies delivered in their clinical work settings were also mentioned [28, 32].

#### Teaching EBP principles

A focus on teaching the six EBP steps was evident in the studies by André et al. [28], Cader et al. [29] and Malik et al. [31]. Research utilisation was emphasised in these reports. There was a vague focus on EBP principles in three of the studies [30, 32, 33] and integrated teaching activities to teach clinical strategies were described in four of the studies [28, 29, 31, 32].

#### Clinical integration and collaboration

In the study by Malik et al. [31], students participated in clinical projects and analysed data with researchers. Clinical experts were also engaged in the lectures.

Cader et al. [29] emphasised the benefit of students carrying out analyses of health needs through collaboration within the clinical practice context. To this end, nursing students conducted ‘mini’ research projects including an analysis of the health needs of a particular patient group with a common problem or diagnosis. Although the nursing students found the assignment challenging and time consuming, they also considered it meaningful because accessing information about health needs made the evidence relevant.

Mattila and Eriksson [32] outlined a learning assignment conducted during a six-week clinical practice period in which students chose topics aimed at utilising research and enhancing their competence in the clinical practice context. The clinical instructor approved a selected research article that was applicable to clinical practice and the nursing students orally presented their findings to fellow students and staff at their clinical practice placement.

A Norwegian pilot study by André et al. [28] focused on participation and cooperation in clinical research projects, which nursing students specified was a motivation for learning EBP. These students strongly appreciated working with experienced nurses on their clinical projects.

### Learning outcomes

Students expressed that writing assignments helped them understand the research process. Based on the nursing students’ reports, they were motivated by being able to choose topics that were of interest to them [29, 32, 34]. Learning outcomes from teaching strategies were presented in most of the studies we evaluated, and it was from these outcomes that the Enhancing analytical skills and Increased awareness of using research subthemes were identified.

#### Enhancing analytical skills

Nursing students reported learning enhanced analytical and critical thinking skills, and some of the findings were outcomes of specific assignments and teaching strategies [28–30, 34]. Students experienced learning outcomes and thus acknowledged the importance of research utilisation to their future clinical practice. It was emphasised in the reports that these students considered their key roles to be research consumers rather than producers [28–30, 32, 34]. Students also developed a greater awareness of the core role of nursing and that use of research is imperative in the nursing profession.

Based on the assignments they were given, the students in these studies reported learning outcomes such as understanding how to apply relevant evidence to everyday clinical practice. In this way, they learned to link research to health needs [28–30]. Mattila and Eriksson [32] reported that nursing students gained greater insight into their future profession. That academic presentations and discussions inspired them to search for research was considered ‘meaningful’.

Although nursing students considered themselves prepared to use research, Friberg and Lyckhage [30] emphasised that students are insufficiently skilled to assess research critically. This perspective is consistent with the findings by Cader et al. [29] that there is a need for further support for developing students’ critical evaluation skills.

Some students emphasised the importance of bringing together clinical practice, their own practical experiences and the research context. They experienced EBP as a platform to facilitate the development of their curiosity and critical reflection within clinical practice [28].

#### Changing attitudes toward utilising research

Nursing students reported research awareness as a learning outcome associated with information gathering and improved information literacy skills [29, 30]. Despite completing acourse, students in one study stated that they had neither a comprehensive understanding of the information literacy concept nor improved skills [33]. Computer and information literacy skills apparently vary among both lecturers and students [33, 35]. However, in several of the studies, increased awareness and understanding of research appeared to be an important learning outcome of information literacy teaching strategies for nursing students [29, 30, 32, 34].

Generating an awareness of how to critically evaluate research evidence rather than producing research is necessary for implementing EBP. To obtain this awareness, it is crucial to find creative ways of guiding undergraduate nursing students to find and critically appraise research reports [30]. These studies emphasised nursing students’ increased awareness that implementing nursing research in clinical practice is a prerequisite to providing safer patient care [28–30, 32].

### Barriers

Barriers to acquiring EBP and research utilisation skills were divided into two subthemes: i.e., information literacy skills and knowledge, and Challenging collaborations.

#### Information literacy skills and knowledge

Discontinuity of information literacy content throughout the curriculum seems to constitute a barrier to searching for and finding research [33]. In addition, some academics reported their own limited awareness of EBP teaching strategies [31].

Nursing students reported finding it challenging to find and interpret research. They were dependent on assistance from librarians and lecturers [32, 33]. The need for more interdisciplinary support to teach information literacy skills was also emphasised in several studies [29, 32, 33, 35]. When integrating EBP, it was challenging for academic nurses to implement innovative teaching strategies because they lacked knowledge, had a large workload or had insufficient time and resources to study new strategies [31].

#### Challenging collaboration

In one study, group work was interpreted as a barrier to learning EBP [34], which demonstrated that dysfunctional group dynamics can negatively affect the learning process. In contrast, in the same study, some students reported positive teamwork experiences that were motivating and enhanced their learning process [34]. In the study by Malik et al. [31], the academic educators reported that their students loved workshops on searching databases. Some nursing students reported that their clinical practice status made it difficult to gather the information required for their assignments [29].

## Discussion

The findings from this initial review demonstrate that various interactive teaching strategies have been emphasised to enhance knowledge and utilise research. However, despite being recommended strategies [3, 14, 36], factors such as teaching strategies that include clinical activities to develop EBP knowledge and skills seem to be given a lower priority. This review identified that self-reports and evaluations show that nursing students report development of critical thinking skills as a learning outcome of various teaching strategies [28–30, 34], which is consistent with earlier studies [16–18]. In contrast, barriers to enhancing students’ EBP knowledge and skills included a weak understanding of information literacy and difficulties finding and interpreting research.

### Interactive teaching strategies

All analysed studies herein reported at least some use of interactive teaching strategies. Patient safety and quality of care in Western society require that future nurses have EBP knowledge, which means that they must use available research as well as patients’ preferences and their own clinical expertise in decision-making processes [14, 36]. We identified studies that emphasised teaching strategies specifically aimed at finding research, critical appraisal and research utilisation through interactive methods [30, 32, 33]. However, it may be challenging for future nurses to obtain sound EBP knowledge if teaching strategies are mainly focused on research utilisation. A commission of health-care professionals and academic leaders presented their vision and common strategy toward strengthening global health-care systems, which argued that cross-professional collaboration in education is a powerful instrument for improving health-care outcomes [37]. Guiding principles, such as the code of ethics, the EHEA framework for expected learning outcomes [20, 21] and health legislation emphasise wider use of the best research evidence in nursing practice, which may explain why teaching strategies are primarily directed at research utilisation. Information literacy skills are important to EBP; however, studies show that nurses and nursing students lack these skills [17, 18, 38–40].

### Clinically integrated teaching strategies

In the present review, three studies [28, 29, 32] focused on clinically integrated teaching strategies in particular. The relationships between the clinical practice context and health needs analysis [29] were emphasised in a six-week clinical practice assignment, using oral presentation as a learning activity [32] and participation in clinical research projects [28].

Ryan [10] identified that learning EBP would have greater relevance for students if teaching strategies took place in a clinical setting. According to Llasus et al. [38], knowledge translation from education to clinical practice is challenging. These authors argue that if nursing students are expected to be able to implement EBP in clinical practice, they must have both EBP knowledge and EBP ‘readiness’, which requires strengthening their confidence in EBP.

Phillips and Cullen [13] observed that development of EBP skills for RN-to-BSN students was influenced by exposure to educational partnerships, contextual teaching and learning, and clinical practice experience. However, the findings from a Norwegian study in physiotherapy students reported a lack of both EBP culture and role models in their clinical practice [41].

A systematic review showed that EBP knowledge in medicine is increasing, irrespective of whether or not it is provided at undergraduate or postgraduate levels. Indeed, learning outcomes appear more effective if the teaching strategies are connected to clinical practice [42]. This notion was emphasised in the core clinical evaluation criteria developed in the Delphi Study by Bostwick and Linden [43]. In contrast, Ilic and Maloney [44] found no difference in learning outcomes. Despite the variety of teaching strategies across the studies we reviewed, they cumulatively show good evidence that any form of teaching EBM increases knowledge.

The findings from this review demonstrate that collaboration through clinical practice and patient care appears to be a relatively low priority. Patients’ preferences are not explicitly considered, despite an increased focus on seeing the patient as a collaborative partner in the EBP paradigm, ethical guidelines and legislation on education and health [2, 3, 20, 21]. This is also contrary to recommendations about the factors that influence EBP skills, such as contextual teaching and learning and practical experience opportunities [13].

### Becoming more analytical

It is worth mentioning that the nursing students in some of the studies included in this review increased their analytical skills because of EBP teaching strategies [28–30, 34], regardless of whether the teaching focused exclusively on interactive or targeted both interactive and clinical strategies. However, research has shown that nursing students have inadequate knowledge to make them capable of judging, reflecting on and critically assessing research [10]. Becoming more analytical and changing attitudes towards utilising research in clinical situations may be essential for nurses in their future careers and could contribute to increased patient safety. These skills may lead to nurses with a higher level of analytical skills and clinical judgment, who have a greater ability to reflect and reason.

### Course assignments as a teaching and learning strategy

According to the review findings, a variety of course assignments promote EBP knowledge and skills. Choosing topics of interest to students motivates them to develop EBP knowledge [29, 32, 34]. Several studies argue that assignments are essential for self-directed, continuous learning [18, 38–40].

### Methodological limitations

There are some limitations to this review. We used a relatively small sample of articles and excluded non-English language studies, which may have caused us to overlook some studies on enhancing EBP skills and knowledge in nursing education. However, to ensure a systematic search process, the literature search was performed by AN, an academic librarian. In addition, our use of several databases likely decreased the possibility of selection bias. The six qualitative studies included in this study were homogeneous in terms of their qualitative research design and meeting our inclusion criteria. Qualitative data from the mixed-methods study that addressed our research question was also included. Variations such as cultural diversity and differences in participant perspectives may also have affected the analyses in these studies. Despite these limitations, we met our goal of examining teaching strategies, learning outcomes and barriers in undergraduate nursing education, from the perspectives of both educators and students.

## Conclusions

Insufficient attention has been paid to the use of EBP principles in nursing education. The teaching strategies identified in the represented studies show that interactive teaching strategies are used alongside traditional lectures to enhance research utilisation skills in nursing education. However, collaboration with clinical practice to enhance EBP knowledge was only vaguely addressed in most of these studies. In conclusion, there is a need to improve educators’ consciousness of and competences in teaching EBP principles, which involves using interactive and clinical integrated teaching strategies. Only seven studies met criteria for inclusion in this review, indicating that further targeted qualitative research is needed.

## Abbreviations

BN: Bachelor Nurse
CASP: Critical Appraisal Skills Programme
EBM: Evidence-Based Medicine
EBP: Evidence-Based Practice
EHEA: European Higher Education Area
IL: Information Literacy
PICo: Population Interest Context
PRISMA: Preferred Reporting Items for Systematic Reviews and Meta-Analysis
RN-to-BSN: Registered Nurses and Bachelor of Science in Nursing
UK: United Kingdom
